# Canine adenovirus type 1 causing neurological signs in a 5-week-old puppy

**DOI:** 10.1186/s12917-019-2173-5

**Published:** 2019-11-21

**Authors:** Samuel J. Hornsey, Hélène Philibert, Dale L. Godson, Elisabeth C. R. Snead

**Affiliations:** 1 Department of Clinical Sciences, Western College of Veterinary Medicine, University of Saskatchewan, Saskatoon, SK S7N 5B4 Canada; 2 Department of Veterinary Pathology, Western College of Veterinary Medicine, University of Saskatchewan, Saskatoon, SK S7N 5B4 Canada; 3 Department of Veterinary Microbiology, Western College of Veterinary Medicine, University of Saskatchewan, Saskatoon, SK S7N 5B4 Canada

**Keywords:** Canine infectious hepatitis, Canine Adenovirus, Canine Adenovirus type 1, Neurological signs, Canine

## Abstract

**Background:**

Infectious canine hepatitis is a rarely encountered disease, that is caused by Canine Adenovirus-1. Clinical signs can vary dramatically, and neurological signs are rarely seen. Neurological manifestation of this disease is rarely reported in the veterinary literature.

**Case presentation:**

A 5-week-old, male entire Husky cross puppy presented for a one-day history of abnormal neurological behaviour (circling, ataxia, vocalization and obtund mentation). The puppy was euthanized shortly after presentation due to rapid deterioration. Histopathology raised concerns for Canine Adenovirus 1 (CAdV-1) based on vasculitis in the brain and intranuclear inclusion bodies in endothelial cell and hepatocytes; immunohistochemistry on brain tissue confirmed CAdV-1 infection.

**Conclusions:**

This report discusses possible routes of infection and manifestations of adenovirus infections causing neurologic signs. It also provides a timely reminder that CAdV-1 should be considered a differential in unvaccinated dogs that present with neurological signs. Further studies are required to better understand the neurotrophic tendencies of this virus.

## Background

Infectious canine hepatitis is a rarely encountered disease in North American domestic canines and is caused by Canine adenovirus-1 (CAdV-1). It has a worldwide distribution and is spread by direct or indirect contact with infected urine, faeces, saliva and respiratory secretions [1]. It has also been reported in populations of wild canids including coyotes, foxes, wolves as well as in bears [2]. Clinical signs only develop in a small number of infected animals, and typically present between day 4 to 9 following exposure [3]. Clinical signs can vary dramatically; they may include pyrexia, inappetence, lethargy, vomiting, diarrhoea, and abdominal pain [1, 4]. Tonsillitis, conjunctivitis, and corneal oedema (classical blue eye) can also be seen. Rarely, neurological signs can result and may include altered mentation, ataxia and seizures [1]. Neurological manifestation of the disease is typically the main clinical signs seen in wild animals [2]. Infected patients may rapidly deteriorate within days from an acute infection or develop a chronic form that leads to hepatic failure and death over weeks to months [3, 5]. Infected animals may also show minimal to no clinical signs and make a full recovery [3]. Protective immunity can be obtained through maternally derived antibodies or vaccination against Canine adenovirus type 2 (CAdV-2) that has been shown to provide protective immunity against CAdV-1 [6].

In this report, we describe a rare case of infectious canine hepatitis associated with neurological abnormalities in a puppy. Necropsy results were suggestive of CAdV-1 infection, which was confirmed with immunohistochemistry.

## Case presentation

A 5-week-old, male entire Husky cross puppy presented to the University of Saskatchewan’s, Veterinary Medical Centre (VMC) emergency service with a one-day history of abnormal neurological behaviour that included circling, ataxia, vocalization and an obtund mentation. On presentation the puppy had an inappropriate mentation, menace was absent bilaterally, normal pupillary light response was noted, a gag reflex was present and no other cranial nerve deficits were noted. The puppy was circling to the left and had an ataxic gait. There were no other significant neurological findings. Neuroanatomical localization was suggestive of multifocal central nervous system lesions within the forebrain and brainstem.

The local humane society had received the dam and her 8 puppies from Northern Saskatchewan; the mother was vaccinated on arrival to the shelter. The puppies were subsequently vaccinated seven days later with a modified live vaccine (Nobivac 1 DAPPC, Merck Animal Health) after all puppies tested negative for canine parvovirus with an enzyme-linked immunosorbent assay (SNAP Parvo test, IDEXX Laboratories, Markham, ON). All the other puppies and the dam were asymptomatic with the exception of one litter mate who presented to the VMC one day prior to the puppy reported on here with a 3-day history of lethargy, increased respiratory effort and bilateral serous nasal discharge. An upper respiratory tract infection was suspected. Amoxicillin (Apotex Inc.; Toronto; ON) 22 mg/kg PO q12 for 10 days was prescribed and the puppy was discharged with instructions to be immediately weaned from the mother and isolated from the other puppies. No neurological abnormalities were noted in this or in any of the other littermates at any time point.

This puppy was placed in isolation on arrival based on a suspicion of an underlying infectious disease, including possible canine distemper. Consent was obtained from the local humane society to perform diagnostics and provide supportive care. Normosol R (Hospira, Montreal, QC) fluids were given intravenously at 4 ml/kg/hr. and analgesia was provided with hydromorphone (Sandoz Inc., Boucherville, QC) 0.05 mg/kg IV q4. An emergency panel revealed a packed cell volume of 22% (26.5–35.5), total protein of 5.8 g/dl (3.7–4.8), Azotstick® (Siemens Healthcare Diagnostics Inc., Tarrytown, NY) blood urea nitrogen of 5-15 mg/dl (13.1–46.2) and blood glucose of 10.3 mmol/l (6.7–8.9). A complete blood count was also submitted to Prairie Diagnostic Services Inc. (PDS), which revealed a moderate regenerative anaemia; red blood cell count (RBC) 3.25 × 10^12^/L (5.8–8.5) and haematocrit (HCT) 0.22 L/L (0.39–0.56). A moderate left shift with toxic change indicative of acute inflammation was also noted; white blood cell count (WBC) 10.7 × 10^9^/L (4.9–15.4), segmented neutrophils 8.0 × 10^9^/L (3.0–10.0) and bands 1.0 × 10^9^/L (0.0–0.1). Bloodwork results included age specific reference ranges relevant for this patient [7].

Initial differential diagnoses for the neurologic signs included canine distemper, bacterial meningitis, protozoal meningitis (e.g., toxoplasmosis), and less likely a possible unusual manifestation of rabies. Symptomatic treatment with broad spectrum antibiotics was initiated and included Metronidazole (Baxter, Mississauga, ON) 25 mg/kg IV q12, Piperacillin (SteriMax Inc., Oakville, ON) 40 mg/kg IV slowly over 30 min q6, and the intent to administer Clindamycin (Intervet, Kirkland, QC) 12.5 mg/kg PO q12. Clindamycin was unable to be administered as the puppy quickly lost his gag reflex as his mentation progressively deteriorated within the first 4 h following presentation. The puppy was losing and regaining consciousness every minute and became non-responsive to external stimuli. After further discussion with the local humane society it was decided for welfare reasons and a suspected poor prognosis to euthanize the puppy. The puppy was euthanized with intravenous Pentobarbital (Bimeda-MTC, Cambridge, ON) 2 ml/4.5 kg; death was confirmed by cardiac auscultation. No additional anaesthetic agent was required due to the puppy’s obtund mentation. The puppy was euthanized 18 h after clinical signs were first noted.

A necropsy was performed at Prairie Diagnostic Services (PDS), Saskatoon, Saskatchewan the following day. The puppy was in good body condition. Significant gross findings were oedematous, mottled pink to pale red lungs, and a diffusely enlarged, pale brown liver. No oedema of the gall bladder was noted.

**Fig. 1:**
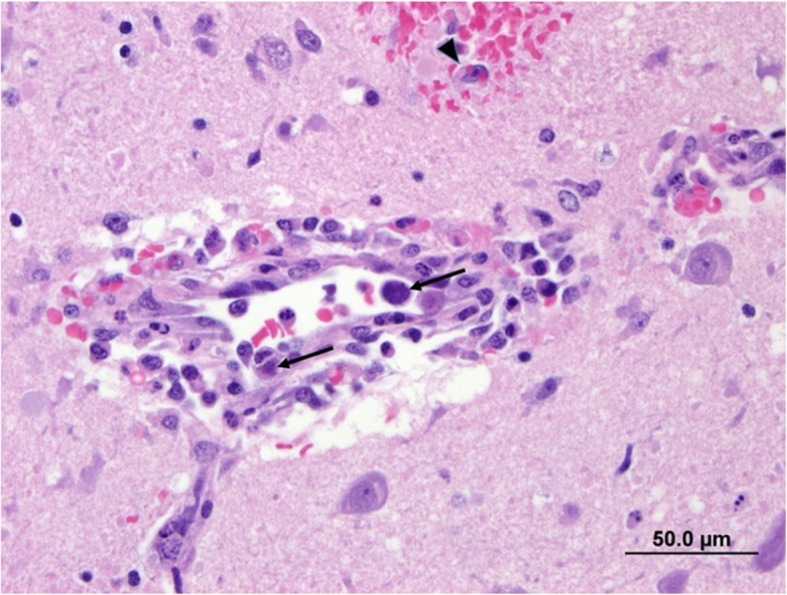
Photomicrograph of vessel in the brain. The wall is infiltrated by macrophages with perivascular accumulation of red blood cells, small amount of fibrin and macrophages. Small haemorrhage in the neuropil and erythrophagocytosis (arrowhead). Endothelial cells contain large basophilic intranuclear inclusion bodies (arrow). H & E stain. Bar = 50.0 μm

Histopathology was performed on major organs including brain (cerebrum, thalamus, cerebellum, pons, medulla), lungs, heart, liver, spleen, eyes, bone marrow, kidneys, lymph nodes, and small intestines. Areas of hyper-cellularity in the brain centered on vessels and accompanied by acute haemorrhage were observed particularly in the corona radiata, caudate nucleus, thalamus, pons and leptomeninges. Vessels were surrounded and infiltrated by macrophages and this was associated with oedema, small amount of fibrin, necrotic inflammatory cells and haemorrhage in the Virchow-Robbin space and the adjacent neuropil. A few macrophages displayed erythrophagia. Endothelial cells were hypertrophied and often contained a large basophilic intranuclear inclusion body (Fig. 1). Hepatocytes were diffusely, mildly vacuolated and there was rare individual cell necrosis. Hepatocyte nuclei also frequently contained large basophilic inclusion bodies. Pulmonary alveoli were filled with oedema fluid, a small amount of fibrin, red blood cells and the alveolar walls were multifocally infiltrated by macrophages. Rarely endothelial cells of interalveolar capillaries contained intranuclear inclusion bodies. Endothelial cells containing these intranuclear inclusion bodies were also commonly seen in vessels of multiple organs including in the kidneys, bone marrow, lymph nodes, liver and retina. Signs of vascular injury and haemorrhage were variably present. The main final histologic diagnoses were meningoencephalitis with vasculitis and hepatic necrosis suspected to be from a viral infection based on the presence of the intranuclear inclusion bodies. The etiological differential diagnoses were Canid Alphaherpesvirus 1 (CaHV-1), Canine Adenovirus type 1 (CAdV) and canine distemper virus (CDV) infections. Given the puppy was older than 3 weeks of age, Canine herpes virus infection was considered less likely.

Immunohistochemistry for Adenovirus, CDV and Rabies were performed on brain tissue. Results were positive for Adenovirus, and negative for CDV and Rabies. CDV was, however, detected by reverse transcription PCR (IDEXX Laboratories) in a sample of whole blood collected prior to euthanasia.

**Fig. 2:**
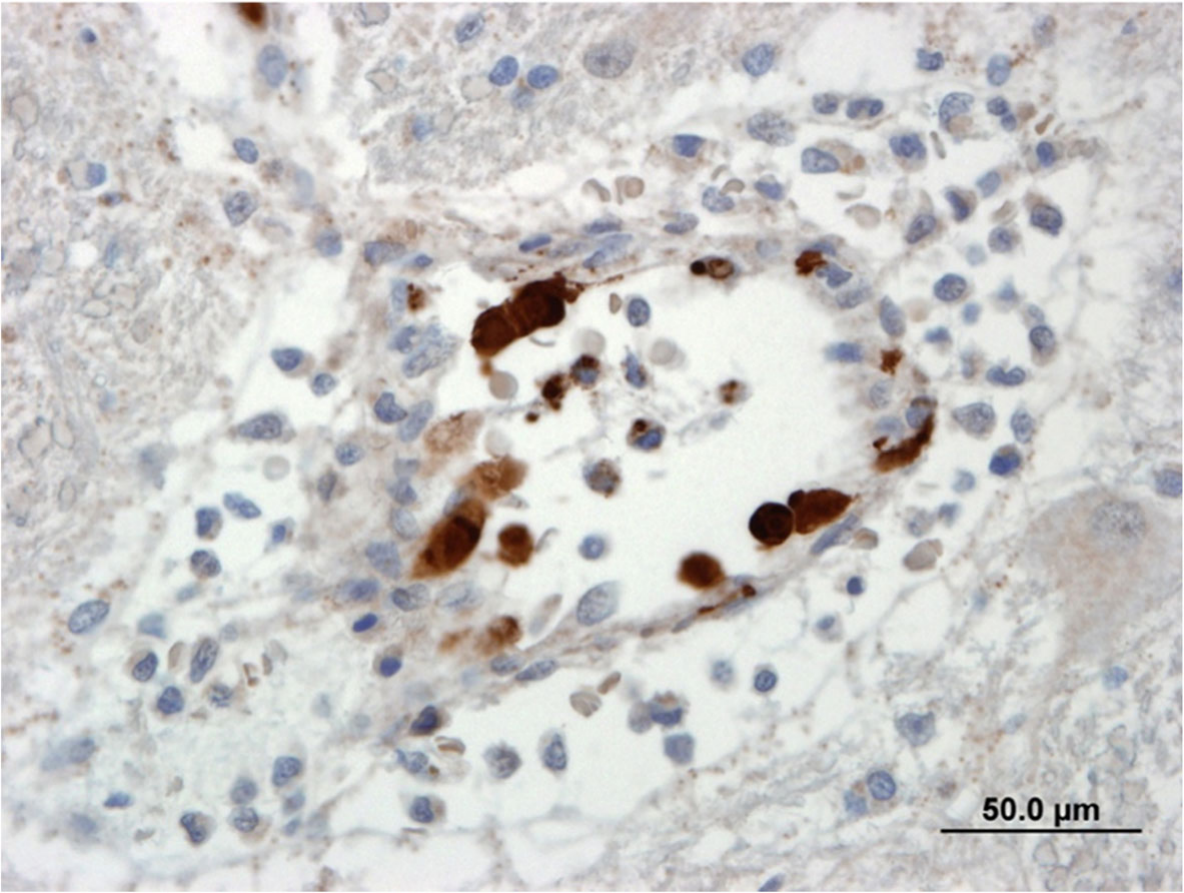
Endothelial cells in the brain with positive immunoreactivity for adenovirus. Strong intracytoplasmic and intranuclear staining. Avidin-Biotin immunoperoxidase. Bar = 50.0 μm

The immunohistochemical staining for both CAdV and CDV was performed on brain tissues at PDS on an automated staining platform (Autostainer Plus, Dako Canada Inc., Mississauga, ON). Heat-induced epitope retrieval was performed, and the primary antibodies (goat anti-CAdV, Virostat, Portland, ME and mouse anti-CDV (clone DV2–12), Custom Monoclonals International, West Sacramento, CA) were used at a 1:4000 dilution. An avidin/biotin blocking reagent (Vector Labs; Burlingame, CA) was applied before the CAdV antibody. Binding of the CAdV antibody was detected using rabbit anti-goat immunoglobulins (Vector Labs; Burlingame, CA) and an avidin-biotin immunoperoxidase complex reagent (Vector Labs; Burlingame, CA), and binding of the CDV antibody was detected using an HRP-labelled polymer detection reagent (EnVision+ System - HRP Labelled Polymer, Dako Canada Inc., Mississauga, ON). The staining was visualized using 3,3′-diaminobenzidine tetrahydrochloride (DAB) (Dako Canada Inc., Mississauga, ON) as the chromogen. CDV antigens were not detected. However, numerous cells with strong cytoplasmic and nuclear staining for CAdV antigens were observed within the endothelial cells, confirming infectious canine hepatitis (Fig. 2.) Further immunohistochemistry staining was also performed at the University of Minnesota Veterinary Diagnostic Laboratory to perform specific staining to differentiate between CAdV-1 and CAdV-2. Results indicated few endothelial cells had positive immunoreactivity for CAdV-1. IHC staining for CAdV-2 was negative.

Follow-up of the litter revealed that none of the 7 other puppies developed neurological signs and the puppy with respiratory signs made a full recovery on antibiotics. The mother and puppies were kept for surveillance at the humane society for 2 months before being adopted out. Recheck of all puppies 5 months later revealed all puppies were doing well, with no current health concerns.

## Discussion and conclusions

This report describes a case of CAdV-1 causing neurological signs in a 5-week old puppy, confirmed by immunohistochemistry on brain tissue. CAdV-1 is a virus that typically infects endothelial cells and hepatocytes, leading to oedema, serosal haemorrhage, and hepatic necrosis [8]. A haemorrhagic diathesis, nephritis and respiratory distress from either laryngitis, tracheitis or less frequently pneumonia may also be seen. Neurological signs are typical of adenoviral infection in wild animals [2] but in domestic dogs neurological manifestations are rarely reported [9–11].

Dogs that die in the acute phase of CAdV-1 causing infectious canine hepatitis typically have an enlarged pale, yellow, mottled liver with patchy areas of necrosis, haemorrhages on abdominal surfaces and in lymph nodes, ascites and gallbladder oedema which is a common finding [1]. The expected microscopy change is hepatic necrosis in centrolobular region and haemorrhages in organs secondary to vascular damage. In this case, the expected gross lesions in the liver and gallbladder were not present. It is possible that this puppy was euthanized prior to these lesions developing.

Neuroanatomical localization was suggestive of multifocal lesions within the forebrain due to changes in mentation and circling. It is suspected that a lack of a menace response bilaterally was due to the patients age; this learned response typically does not develop until 10–12 weeks of age [12]. But we cannot rule out that its absence is the cause of disease within the central nervous system, particularly the forebrain.

The source of exposure to CAdV-1 in this case was not determined; however, we can hypothesize several possible routes of infection. Given the mother was unvaccinated prior to arriving at the shelter, she may have acted as an inapparent carrier of the virus. The virus can be shed in the urine for a least 9 months post-infection [4]. It is also possible that the puppies and the dam were all exposed to the same environmental source of the virus, either in Northern Saskatchewan where they were rescued from, or exposure may have occurred at the local human society. Infectious canine hepatitis is known to be present in wild dog and mammal populations in Canada [13–16] and the virus can remain viable at temperatures below 4C for months so environmental exposure in Northern Saskatchewan is very possible [4]. If exposure was recent for the dam then no maternally derived antibodies would have been transferred to the puppies through the colostrum leaving them all vulnerable to infection [1]. Since only one puppy was affected clinically it maybe that this puppy did not get any colostrum and so potentially was the only puppy vulnerable to infection if the dam and litter were exposed to a common source. The reason why only one puppy out of a litter of 8 developed neurological signs is unknown. In another reported case of neurological manifestation of this disease, 9 out of 11 puppies developed neurological signs [9]. It is possible that this individual puppy was the only one vulnerable in the litter from either failure of passive transfer, if he did not receive colostrum at birth, or perhaps if he was unable to develop immunity to the virus due to another underlying concurrent condition or possible congenital immunodeficiency.

There was also the possibility that an infection with CAdV-2 not CAdV-1 was responsible for the neurologic signs in this puppy. CAdV-2 typically causes infectious tracheobronchitis in dogs but it has been suggested that CAdV-2 has neurotrophic tendencies in the mammalian brain [17] and there is also one report of CAdV-2, discriminated and confirmed by PCR from CAdV-1, causing acute neurological signs and death in 4 puppies [18]. Another report has described two fatal cases of infectious canine hepatitis in which after genomic testing, the strain of CAdV-1 had more genomic similarities to CAdV-2 [19]. Initially it was postulated that recombination of CAdV-1 and CAdV-2 may have occurred, leading to either vaccine failure or increased virulence. However, after specific immunohistochemistry staining for CAdV-1 and CAdV-2 at the University of Minnesota Veterinary Diagnostic Laboratory, results confirmed a positive stain for CAdV-1, with staining for CAdV-2 being negative.

Initially there was concern for canine distemper virus infection based on the litter mate presenting for upper respiratory tract signs and due to this puppy subsequently presenting with neurologic signs. Genomic RNA of CDV was detected in the blood by RT-PCR; however, viral antigens were not detected in the brain or other tissues by immunohistochemistry. In this case it is likely that the molecular test for CDV on whole blood identified a vaccine strain due to recent vaccination with a modified live virus and the PCR test identified the replicating vaccine strain of the virus [20]. A recently developed test, the duplex reverse transcription PCR could have been performed as it has been reported to be able to differentiate between the wild type and vaccine form of CDV by quantification [21]. Unfortunately, this test can only be performed on respiratory swabs so was not an option here but with more foresight could possibly have been performed on the littermate of this puppy. It is important to test for CDV when suspicious of CAdV-1 infection, as multiple studies have reported co-infection of the two viruses, which can increase the mortality rate [22–24]. A post-vaccinal CDV infection has also been previously reported [25] and could have been a possible differential diagnosis for the neurologic signs in this puppy but this was ruled out by the lack of IHC for CDV in the brain tissues.

Other initial differentials for this puppy included rabies, CaHV-1 infection and a protozoal disease. IHC staining for rabies was performed on brain tissue, results of which were negative. IHC testing for CaHV-1 is not available. But given the positive IHC staining for CAdV-1 within brain tissue, the postmortem findings are likely attributed to CAdV-1 and not canine herpes virus. There were also no findings on postmortem of protozoal disease such as toxoplasma or neospora.

This case report describes a rare neurological manifestation of CAdV-1 infection in a 5-week-old puppy that was confirmed by immunohistochemistry. This report demonstrates that as clinicians, we should consider infectious canine hepatitis as a possible cause for neurological signs in a young unvaccinated dog. It also reinforces the importance of vaccination in preventing this disease, and that sensitive detection tests such as molecular tests following the administration of modified live vaccines may confound interpretation of test results for Canine Distemper.

## Data Availability

The datasets used and/or analysed during the current study available from the corresponding author on reasonable request.

## Abbreviations

CAdV-1: Canine adenovirus 1
CAdV-2: Canine adenovirus 2
CDV: Canine distemper virus
CAdV: Canine Adenovirus
IHC: Immunohistochemistry
PCR: Polymerase Chain Reaction
RBC: Red blood cell
HCT: Haematocrit
WBC: White blood cell
